# The Peptide Vaccine of the Future

**DOI:** 10.1074/mcp.R120.002309

**Published:** 2021-02-08

**Authors:** Annika Nelde, Hans-Georg Rammensee, Juliane S. Walz

**Affiliations:** 1 Clinical Collaboration Unit Translational Immunology, German Cancer Consortium (DKTK), University Hospital Tübingen, Tübingen, Germany; 2 Department of Immunology, Institute for Cell Biology, University of Tübingen, Tübingen, Germany; 3 Cluster of Excellence iFIT (EXC2180) “Image-Guided and Functionally Instructed Tumor Therapies”, University of Tübingen, Tübingen, Germany; 4 German Cancer Consortium (DKTK) and German Cancer Research Center (DKFZ), Partner Site Tübingen, Tübingen, Germany

**Keywords:** Peptide vaccine, HLA ligands, Cancer vaccination, Adjuvants, Combination therapy, Mass spectrometry, Biomarkers, APC, antigen-presenting cell, HLA, human leukocyte antigen, TLR, Toll-like receptors

## Abstract

The approach of peptide-based anticancer vaccination has proven the ability to induce cancer-specific immune responses in multiple studies for various cancer entities. However, clinical responses remain so far limited to single patients and broad clinical applicability was not achieved. Therefore, further efforts are required to improve peptide vaccination in order to integrate this low-side-effect therapy into the clinical routine of cancer therapy. To design clinically effective peptide vaccines in the future, different issues have to be addressed and optimized comprising antigen target selection as well as choice of optimal adjuvants and vaccination schedules. Furthermore, the combination of peptide-based vaccines with other immuno- and molecular targeted therapies as well as the development of predictive biomarkers could further improve efficacy. In this review, current approaches in the development of peptide-based vaccines and critical implications for optimal vaccine design are discussed.

## Highlights

• Several prerequisites are mandatory for clinical effective peptide-based vaccines
• Overview of current approaches regarding antigen selection and adjuvants
• Suitable combinations will enhance the efficacy of peptide vaccines in the future
• Biomarkers could be useful to preselect eligible patients for vaccination

## Graphical Abstract

**Figure:**
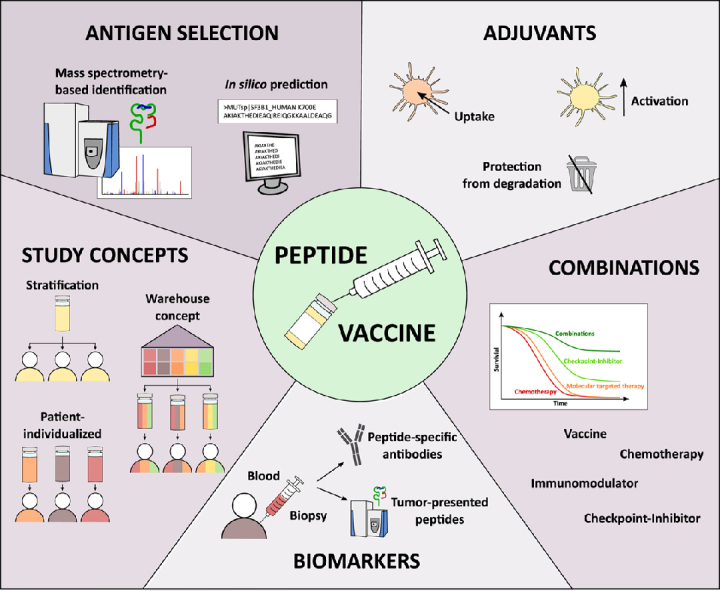

## In Brief

Therapeutic peptide-based vaccination approaches for the treatment of cancer patients have shown first glimmers of success. However, to achieve broad clinical efficacy and implement peptide vaccinations in the standard treatment of cancer patients future peptide vaccines need further optimization in terms of target antigen selection, adjuvant choice, vaccination schedules, delivery routes, biomarkers, and combinatorial drugs.

## Peptide-Based Vaccination

The idea of activating the immune system to combat cancer cells was already documented in ancient Egypt (1) and was then again pursued in the late 19th century by William Coley, who had been exploiting streptococcus-mediated tumor rejection (2, 3), probably after hearing from similar experiments by Wilhelm Busch (4). Such antigen-independent strategies build the cornerstone for a broad and promising research area aiming to actively induce antitumor T cell responses using various different strategies. Today, multiple antitumor immunotherapy approaches (5), including immune checkpoint blockade, CAR T cells, and antibody-based therapies, have found their way into the clinic and even established in the routine of anticancer treatment. Therapeutic cancer vaccines, such as protein-, peptide-, DNA-, RNA-, and dendritic cell– or tumor cell–based vaccines (6, 7), are designed to generate new or amplify preexisting antigen-specific T cell responses against malignant cells. Peptide-based vaccines represent a low-side-effect vaccination approach using synthetic tumor-associated or -specific peptides or peptide combinations that are designed to induce and activate peptide-specific tumor-reactive T cells *in vivo*. These peptides are presented on the surface of cells on human leukocyte antigen (HLA) molecules and are recognized by the T cell receptor of CD4^+^ and CD8^+^ T cells.

## Antigen Selection—The Devil Is in the Details

The most essential aspect for a clinically effective peptide vaccination is indeed the selection of optimal antigen targets, which should exhibit a highly frequent and tumor-exclusive presentation on the surface of tumor cells and must be capable of recognition by the patients’ T cells. In recent years it has become apparent that neoepitopes derived from tumor-specific nonsynonymous somatic mutations are the central specifications of immune checkpoint inhibitor-induced T cell responses (8). This is in line with the correlation of response to checkpoint inhibitor therapy with tumor mutational burden (9, 10). Owing to their true tumor specificity, neoepitopes are not affected by central or peripheral tolerance, which renders them ideal targets. The encouraging potential of neoantigen-based vaccination approaches was already investigated in preclinical and early-phase clinical trials assessing neoantigen-based peptide vaccines in several different cancer entities (11, 12, 13, 14). Yet, substantial challenges remain in the effective identification of immunogenic and naturally presented neoepitopes derived from point mutations, insertion–deletions or frame-shift mutations. Therefore, in recent years, several different bioinformatic tools have been developed to improve the selection and identification of candidate neoepitopes for vaccine design. Based on whole-exome sequencing data algorithms such as NeoPredPipe, MuPeXI, pVAC-Seq, and CloudNeo identify patient-individual mutations, predict the affinity of neoantigen-derived peptides to HLA molecules regarding to the individual patient’s HLA allotypes, integrate tumor mutation and expression data, predict the immunogenicity of these peptides, and evaluate their potential as T cell epitopes (15, 16, 17, 18). Still, this selection and prediction process remains challenging especially for HLA class II–presented neoepitopes recognized by CD4^+^ T cells (19). In addition, the use of mutation-derived vaccine peptides harbors two main issues: the majority of tumor-specific mutations are patient-specific and therefore not suitable for broadly applicable immunotherapy, but rather can be used for personalized approaches. Furthermore, in cancer entities with low mutational burden, the role and impact of neoepitope-directed T cell responses appear to be marginal, as several studies have demonstrated that the frequency of naturally presented HLA-presented neoepitopes is less than 1% of the number of nonsynonymous mutations based on mass spectrometric analysis (11, 20, 21, 22). Mass spectrometry also revealed that hotspot sequence regions within individual proteins generate the majority of HLA-presented peptides in contrast to other protein domains that are not efficiently processed for HLA presentation (21, 23, 24), which highlight the need for immunopeptidomics to select truly processed and naturally presented peptides and neoepitopes. Therefore, future large-scale immunopeptidomics studies may reveal such hotspots in the human proteome leading then to improved bioinformatics tools that prioritize and select neoepitopes based on their location within such hotspot regions.

Besides mutation-derived neoepitopes unmutated tumor-associated self-peptides have gained renewed attention. These unmutated peptides can arise from aberrantly expressed or processed antigens in the tumor cells owing to differential tumor-specific gene expression, posttranslational modification, antigen processing, or other tumor-specific cellular processes (25, 26). For the selection of suitable tumor-associated unmutated peptides different strategies have been applied. For several years, the identification of tumor-associated antigens was based solely on gene expression analyses identifying overexpressed proteins, followed by *in silico* predictions of HLA-presented peptides (27, 28, 29) with algorithms such as SYFPEITHI (30) and NetMHCpan (31). Subsequently, the tremendous number of predicted candidate peptides had to be screened for immunogenicity by extensive *in vitro* assays. A major drawback of such prediction-based approaches lies in the distorted correlation of gene expression with HLA-restricted antigen presentation, since the immunopeptidome is an independent complex layer formed by the antigen presentation machinery and therefore does not necessarily mirror the transcriptome or the proteome (21, 32, 33, 34, 35). In contrast to epitope prediction algorithms, the large-scale and in-depth direct identification of naturally HLA-presented peptides, known as HLA ligandome or immunopeptidome, from primary patient samples by mass spectrometry (36, 37, 38, 39, 40, 41, 42, 43, 44) is the only unbiased approach to comprehensively identify tumor rejection antigen candidates and validate the natural processing and presentation of these antigens (45). For the identification of tumor-associated unmutated self-peptides not presented on benign cells and tissues, the extensive screening of immunopeptidomes derived from benign tissues is of paramount importance. The HLA ligand atlas (www.hla-ligand-atlas.org/) is an open-source tool providing a comprehensive collection of mass spectrometry–identified HLA-presented peptides on benign tissues (46, 47), which has currently been submitted to a peer-reviewed journal. Furthermore, comparative transcriptome and proteome expression profiling between tumor and normal tissues as provided, for example, by the Genotype-Tissue Expression Consortium (48) and the Human Protein Atlas (49), can help to reconsider vaccine candidates and filter out genes and proteins highly expressed in normal tissues. However, the above-described distorted correlation of gene expression, proteome, and immunopeptidome (21, 32, 33, 34, 35) clearly limited the informative value of these analysis. Furthermore, it is important to note that tumor antigens are not always determined at the protein level. The individual peptides can be clearly tumor-associated, even if the source protein itself is not. This can occur through differential processing, posttranslational modifications, or antigen processing in the tumor cell and highlights the need for immunopeptidomic databases of benign tissues and cells.

In addition to the selection of optimal antigens, additional questions rise about the ideal peptide length, the choice of CD4^+^ *versus* CD8^+^ T cell epitopes, as well as the number of different peptides within the vaccine cocktail. In recent years it has become evident that the selection of HLA class II–restricted antigen targets recognized by CD4^+^ T cells in addition to CD8^+^ T cell–recognized HLA class I–presented peptides is of paramount importance for clinical outcome. CD4^+^ T cells harbor a crucial role for effective antitumor immunity (50) and could further improve and sustain T cell reactivity (51) and tumor clearance (52). CD4^+^ T helper cells induce and intensify more permanent tumor immune control (53), epitope spreading (54), CD8^+^ T cell expansion and survival (55), as well as tumor immune cell infiltration (56). In addition, CD4^+^ T cells also exhibit direct antitumor effector function (57). Therefore, the artificial elongation of tumor-associated CD8^+^ Tcell epitopes or the application of multivalent long peptides comprising at the same time CD4^+^ and CD8^+^ T cell epitopes displays promising attempts to improve the efficacy and clinical outcome of peptide vaccination (58, 59, 60, 61, 62, 63). The enhanced induction of T cell responses with long vaccine peptides could also be explained by the fact that exact epitopes bind directly to HLA class I molecules of any somatic cell and thus also to nonprofessional antigen-presenting cells (APCs) without further processing, which could then lead to suboptimal T cell priming and induce T cell tolerance (51, 64, 65). Long peptides, on the other hand, require processing and are therefore only presented by professional APCs (66, 67). We therefore believe that HLA class II–restricted target antigens also in terms of elongated CD8^+^ T cell epitopes are an indispensable component for clinically effective peptide vaccines. This is further supported by the higher promiscuity of HLA class II molecules leading to a not so tight HLA restriction of the presented peptides (68) in contrast to CD8^+^ T cell epitopes. This enables the presentation of the same CD4^+^ T cell epitope on different HLA class II molecules and allows the vaccination of all patients with the same cocktail, regardless of their HLA allotype.

In contrast to other antigen-specific treatments, *e.g.*, CAR T cells, a remarkable advantage of peptide-based vaccines is the possibility of a simple and cost-effective combination of several different tumor-associated antigens in a single injection. Multipeptide vaccines could therefore overcome the issue of antigen loss and reduce the risk of immune escape, which often occurs under therapeutic pressure (69). The approach of multipeptide vaccination is further supported by the observation that patients with immune responses to multiple tumor-associated antigens showed higher levels of disease control and harbor a survival benefit (38, 70).

Furthermore, validation of target antigen expression on patients’ tumor cells prior to the administration of a peptide vaccine to a patient could considerably improve the proportion of patients who can benefit from a specific vaccine. Validation of both unmutated and mutated target antigen expression can be carried out at different levels such as transcriptome, proteome, or immunopeptidome. For mutated antigens, the proof of the presence of the somatic mutation by whole-exome sequencing or RNA-Seq is required, but the additional evidence of the natural presentation within the immunopeptidome is usually rather difficult for these antigens (71). However, for unmutated target antigens the validation of antigen expression using immunopeptidomic analysis is highly recommended, especially in contrast to transcriptomic or proteomic detection of antigen expression, which often shows a distorted correlation to HLA antigen presentation (21, 32, 33, 34, 35).

Besides the presentation of the target antigens on the patients’ tumor cells, the immunogenicity of the individual vaccine peptides is of paramount importance and has to be proven in T cell–based assays prior to inclusion of the specific peptide in a vaccine cocktail (72, 73). Generally, two different approaches can be utilized to assess T cell responses to a specific antigen: 1) *in vitro* priming of naive T cells or 2) *ex vivo* or *in vitro* stimulation of preexisting memory T cells. For the detection of preexisting memory T cell responses patient-derived peripheral blood mononuclear cells or tumor-infiltrating lymphocytes are needed since tumor antigen-specific T cells are normally not abundantly present in healthy individuals. Depending on the abundance of preexisting memory T cells, their detection can be performed directly *ex vivo* or after a short *in vitro* peptide-specific amplification with cytokines such as interleukin 2 (IL-2). As readout different methods can be applied such as enzyme-linked immunospot assay (74), intracellular cytokine staining (75), or multimer staining (76). Such immunogenicity tests should also be performed during a clinical trial to assess the induction of vaccine-induced T cell responses in vaccinated patients.

In recent years, multiple peptide-based vaccination trials have tested different combinations of short (77) *versus* elongated peptides (11), of single peptide–based (78) *versus* multipeptide vaccines (79), or of mutated (11) *versus* unmutated (80) peptides in a variety of different cancer entities. The induction of peptide-specific T cell responses was demonstrated against mutated and self-peptides equally (71) as well as short (77) and long peptides (11). However, patient and antigen selection, dosing, vaccine schedule, as well as usage of adjuvants and combinatorial treatments differ in all of these studies, which hampers a comprehensive comparison and conclusion for the development of improved peptide vaccines.

The chemical or sequence modification of the individual vaccine peptides represents an additional option to improve the efficiency of the vaccine-induced antitumor responses (reviewed in 81, 82). Amino acid substitutions of self-antigens could circumvent tolerance against unmutated self-peptides. Altering the amino acid sequence could not only increase the peptide binding affinity to its respective HLA molecule, but also improve its immunogenicity (83, 84, 85). Besides the impact on the strength of a T cell response, altered peptide sequences could also modulate the character and polarization of the peptide-specific immune responses (86, 87). Especially in chronic diseases such as cancer or viral infections heteroclitic peptides can help to restore T cell function and proliferation (88) and repeal frequently occurring T cell exhaustion caused by antigen persistence (89). However, such changes also harbor the potential risk of weakening the T cell response or potentially inducing the expansion of T cells with irrelevant or even autoreactive specificities. Therefore, the careful evaluation of altered peptide ligands is of paramount importance.

In addition to the modified amino acid composition, direct conjugation of ligands of pattern recognition receptors such as CpG or Pam3CSK4 for Toll-like receptors (TLRs) (90, 91) or C-type lectin receptor–specific mannosylation (92) on long vaccine peptides could enhance antigen uptake and guide intracellular trafficking, thereby favoring antigen presentation.

## Adjuvants and Delivery Mode—It Is All About Activation

For the induction of effective T cell responses through peptide-based vaccines the choice of optimal, strong adjuvants or immunostimulators and ideal delivery routes is of paramount importance to ensure that the vaccine peptides are appropriately sensed by and activate the immune system. The main focus of peptide vaccination is here to induce a type 1–polarized, cell-mediated immunity rather than a type 2–polarized and humoral response (93). For achieving an optimal T cell–mediated antitumor effect, the activation and expansion of antigen-specific T cells is mandatory, which necessarily requires the three well-known signals of T cell receptor stimulation, appropriate costimulation, and specific cytokines (94, 95). Therefore, adjuvants need to deliver the peptide to dendritic cells and activate and mature these APCs to accomplish a solid T cell response. The purpose of such adjuvants thus comprises the protection of the peptide and prevention of immediate degradation, the efficient uptake by APCs, as well as the appropriate and full activation of APCs. Delivery vehicles, which ideally should have a depot effect, can be composed, for example, of oil depots such as Montanide ISA 51 (incomplete Freund’s adjuvant analog) (96, 97). Montanide is therefore mixed with the peptides prior to vaccination to generate a water-in-oil emulsion. For efficient uptake, the peptides can also be encapsulated in structures such as liposomes (98) and nanoparticles (99) or can be covalently conjugated to adjuvants (90, 92). In multipeptide vaccination approaches and especially in patient-individualized approaches the covalent linkage of adjuvants to each individual and single peptide is cumbersome for clinical translation.

So far the cytokine granulocyte-macrophage colony stimulating factor (GM-CSF) that initiates the recruitment, maturation, and activation of dendritic cells (100) has been one of the most common adjuvants applied in anticancer peptide vaccination trials but its adjuvant effect remains weak (71, 101, 102). For activation and maturation of APCs, signaling through TLRs and their ligands is known to induce optimal and strong activation. Therefore, potent adjuvants often mimic such TLR ligands. Especially TLR4 ligands are known to enable a potent activation of APCs (103). However, the prototype among TLR4 ligands—lipopolysaccharide (LPS)—is not sustainable for clinical application owing to substantial toxicity (104, 105). Its chemically detoxified form MPL (3-O-desacyl-4’-monophosphoryl lipid A) (106) is an approved adjuvant, for example, in human papillomavirus vaccines and furthermore investigated in different vaccination approaches (107, 108). The most commonly used TLR agonist (71) poly-ICLC (Hiltonol) is a polyinosinic-polycytidylic acid (poly-IC) stabilized by lysine and carboxymethylcellulose (109), which enhances vaccine-induced T cell responses (110) by TLR3 signaling. Recently, the novel, water-soluble adjuvant XS15, a synthetic TLR1/2-binding Pam_3_-Cys-derivate covalently linked to a single synthetic—nonvaccine—peptide (GDPKHPKSF), was described as an effective vaccine adjuvant inducing unpreceded strong and long-lasting CD8^+^ and CD4^+^ T cell responses in first-in-man proof-of-concept experiments (111). The first clinical trials using XS15, including antitumor (EudraCT 2020-002367-65) as well as highly relevant anti-SARS-CoV-2 peptide vaccinations (EudraCT 2020-002502-75; EudraCT 2020-002519-23), will start recruitment within 2020. Since the induction of the antitumor T cell responses and the clinical outcome has so far been unconvincing, it is mandatory to investigate novel adjuvants like XS15 in clinical trials to further increase the strength and unlock the power of peptide-based vaccines.

Further improvement of imaging technologies to track either the peptide vaccine at the molecular and (sub)cellular level (112) or the peptide-specific T cells in the organism (113) will further expand our knowledge of antigen delivery, uptake, and processing as well as of antigen-specific T cell routes. This knowledge can then be utilized to further improve peptide-based vaccination regarding delivery vehicles, adjuvants, and administration route.

## Treatment Concepts—Broadly Applicable or Personalized?

For the clinical application of immunotherapeutic approaches three different study concepts and strategies comprising distinct levels of personalization of drug products and anticancer therapies have been proposed: 1) stratification, 2) warehouse-based personalization, and 3) individualization (114, 115). Each of these three concepts harbors several advantages as well as disadvantages that need to be considered. Stratification-based treatment decisions as the basic level of personalization are already standard in clinical routine, for example in the biomarker-based application of targeted therapies for specific mutations (116) or antibody-based immunotherapies (117). Stratification selects suitable patients who will benefit from a specific therapy based on the availability of predefined tumor-associated criteria and will then all be treated with one invariant drug product. Only patients harboring the respective tumor feature will receive the therapy. Thereby, stratification represents an approach with an enhancement of treatment efficacy and in the same time minimization of side effects. However, for peptide-based vaccination approaches stratification alone seems not suitable owing to the high intraindividual differences already starting with patient–individual HLA allotypes. Therefore, stratification-based peptide vaccine approaches focus on very common HLA allotypes such as HLA-A∗02 or HLA-A∗24 thereby excluding a substantial proportion of patients (70, 71). Consequently, current vaccine designs are more and more focusing on warehouse approaches (77, 80, 118), patient-individualized concepts (119), or also a combination of both (71). The so-called warehouse concept enables the composition of patient-specific drug products assembled from a collection of predefined and premanufactures high-frequent tumor-associated peptides. Therefore, each peptide within the warehouse is separately manufactured and depending on the patient’s individual characteristics such as HLA allotypes or tumor-presented peptides the peptide vaccine cocktail is then individually assembled using these off-the-shelf peptides. Thus, the warehouse concept enables the individualization of vaccine cocktails in a time- and cost-saving manner. For example, for a hypothetical warehouse peptide vaccination study covering eight different HLA allotypes with five peptides each and including 25 patients, who should be vaccinated with a multipeptide cocktail comprising, for example, as commonly practiced and approved by regulatory authorities (71, 80), 10 peptides, only 40 different peptides have to be produced. The number of 40 peptides is calculated by multiplying the number of unique peptides per allotype by the number of different HLA allotypes covered within the respective warehouse. A warehouse covering, for example, eight of the world’s most common HLA class I allotypes could be used to treat approximately 90% of a patient cohort, which could be calculated for individual allotype combinations using the population coverage tool of IEDB (http://tools.iedb.org/population/) (120, 121).

In contrast, completely individualized peptide-vaccine concepts are based on the selection and on-demand *de novo* production of single patient-specific drug products. The advantage of such approaches is the patient-specific design of such vaccines individually tailored on the patient’s HLA allotypes, single rare mutations, and peptides presented on the individual tumor reaching the maximal drug benefit for each cancer patient. Personalized peptide vaccines can therefore comprise patient-tailored neoepitopes based on individual sequencing data and/or nonmutated tumor-associated peptides based on the patient-individual mass spectrometric analysis of tumor-presented peptides. Therefore, sequencing or mass spectrometry-based identification of patient-individual neoepitopes and nonmutated tumor-associated peptides becomes indispensable. The translation of sequencing data, mass spectrometry–based immunopeptidomics and their combination is already investigated in different clinical trials (71, 80, 122). However, the low-throughput sample capacity is still a major issue. Further limitations of completely individualized approaches are the huge peptide production costs and the limited drug production capacity. The enormous differences in the estimated costs between peptide warehouse approaches and patient-individualized vaccines arise mainly from the increased number of different peptides required for patient-individual approaches. Compared with the warehouse approach, for the hypothetical study with 25 patients and 10 peptides per cocktail a fully individual approach would require the production of in total 250 peptides resulting in more than 5-fold higher peptide production costs.

## Vaccination Schedule—The Right Time Point Also Matters

Besides the selection of the best fitting treatment concept also the schedule and timing of vaccination represent an essential pillar for clinically effective vaccination. The selection of the optimal time point for vaccination in the course of cancer treatment is extremely important. Peptide-based vaccination approaches should ideally be administered in the setting of an intact T cell compartment with an optimal effector to target cell ratio (123). This could be optimally achieved in an adjuvant setting after surgery or in first-line remission, *e.g.*, minimal residual disease induced by standard chemotherapy or radiation (124, 125). Furthermore, for selection of the optimal vaccination time point, potential concomitant therapies must be taken into account as these drugs can influence the outcome and efficacy of peptide vaccination, as discussed below. Furthermore, the vaccination schedule, including primary and boost vaccinations, may have an impact on the effectiveness and duration of the antitumor T cell response. However, there is hardly any systematic comparison of different vaccination regimens and each clinical trial uses different vaccination schedules, thus making evaluation extremely difficult.

## Combinations—It Is Easier Together

Important considerations for clinically effective peptide vaccination encompass not only the actual design and formulation of the peptide vaccine itself, but also furthermore require the awareness of effective combinatorial treatment options. Cancer vaccines alone may be effective in cases of early cancer diagnosis or in the setting of minimal residual disease to prevent relapse or recurrence. However, combinatorial treatments might enable to successfully treat even established cancers as well as to overcome tumor-mediated immunosuppression, immune escape mechanisms, impaired T cell infiltration to the tumor side, or profound immune defects (126, 127, 128, 129). The range of possible combinations is endless, *e.g.*, combinatorial approaches of cancer vaccines with immune checkpoint inhibitors, chemotherapies, radiotherapy, neutralizing antibodies to inhibitory cytokines, small molecule inhibition of regulatory T cells, or immunomodulatory drugs have already been investigated in several studies (80, 130, 131, 132, 133) with the main goal to optimize the T cell compartment. However, combinatorial cancer therapies might also negatively impact T cell responses, as shown in a phase III peptide vaccination study combined with the tyrosine kinase inhibitor sunitinib (134), which failed to confirm the vaccine-induced immune responses and clinical outcome of the corresponding phase II trial testing the peptide vaccine without sunitinib (70). This calls for the extensive preclinical and clinical analysis of the effect of combinatorial drugs on T cell response.

In addition, anticancer drugs can also have marked effects on the immunopeptidome of tumor cells, including HLA surface expression (135), HLA allotype distribution (136), the presentation of vaccine target peptides, as well as the induction of novel, cryptic, treatment-associated ligands (136, 137). Therefore, it is of paramount importance to characterize also the effects of combinatorial drugs not only on the effector cells but also on the antigenic landscape of the target cells (138). Furthermore, the induction of novel, treatment-associated HLA ligands on cancer cells under specific treatments could be utilized for the identification of novel suitable antigen targets that could be eligible as novel treatment-associated targets for combinatorial peptide vaccinations (137, 138, 139).

## Biomarkers—Important to Know

The identification of prognostic factors and predictive biomarkers is very important to assist the selection of those patients who are expected to respond well to peptide vaccination, in the best case even before the start of therapy or at least early after treatment initiation. However, the criteria for patients selected for peptide vaccination and the selection of individual vaccine peptides vary greatly between different clinical trials, including preexisting T cell responses (77), expression of target antigens on the patient’s tumor (71, 80) or on soluble HLA molecules in the plasma (140, 141, 142), peptide-specific preexisting immunoglobulin G responses (77), or individual transcriptome analysis (71). Several studies have reported that the immunoglobulin G response against the vaccine peptides is a potential biomarker for the prediction of overall survival (143, 144). However, the biological role of peptide-specific antibodies remains still unknown. Increased peptide-specific immunoglobulin G levels might reflect the activation of CD4^+^ T cells (144). Further plasma biomarkers, which were found to be useful predictive markers for clinical outcome after peptide vaccination, are the level and integrity of circulating cell-free DNA (145), or specific microRNAs (146). Recently, a study also demonstrated that the prevaccination, but not postvaccination, blood cell composition (high lymphocyte cell counts and percentage) and the inflammatory signature (low levels of CRP, IL-6) are associated with overall survival after personalized peptide vaccination (147). The expression level of the immune checkpoints PD-1 and Tim-3 on T cells has been demonstrated to correlate with overall survival in a peptide vaccination trial (148) further highlighting the importance of combinatorial approaches with immune checkpoint inhibitors.

The identification of biomarkers that can be utilized for the selection of patients and predict the response to peptide-based immunotherapy is both important and attractive. Since multiple factors may influence the induction of clinically effective anticancer T cell responses after peptide vaccination, their predictive and prognostic capabilities have to be validated by further large-scale, prospective, and randomized clinical trials.

## Limitations and Side Effects of Peptide-Based Vaccinations and The Way Out—Little by Little One Goes Far

Although the expectations placed in peptide-based vaccination approaches have not been fulfilled so far and clinical success is still limited, more and more ideas and ways to overcome these disadvantages are emerging. In this section we will discuss the drawbacks of peptide-based vaccine approaches, take a look at the issue of adverse effects, and point out solutions to overcome these disadvantages, which have already been partly applied and evaluated in different clinical trials. A first limitation is the low immunogenicity of vaccine peptides reported in some clinical trials (11), which can be overcome in different ways: optimized preclinical selection of suitable antigens and the characterization of their immunogenicity, the modification and affinity maturation (81, 82), as well as the use of improved strong but nontoxic adjuvants as discussed above are the most important points to address this problem. A further disadvantage is a potential immune escape if vaccination is performed only with single target peptides. Thus, multiple epitope vaccine approaches are already applied in the majority of clinical trials. Furthermore, the HLA allotype restriction of HLA class I–presented peptides might exclude patients with unmatching HLA class I allotypes, which could be overcome by using HLA class II–presented promiscuous CD4^+^ T cell epitopes embedding multiple HLA class I peptides in the vaccine.

Despite these rather peptide-specific limitations, there is in our view a major obstacle for the clinical success of all T cell–based immunotherapies including vaccines, which is the limited accessibility of the tumor for cancer-specific T cells. Especially in solid tumors, T cell recruitment is often impaired by an immunosuppressive tumor environment (149). To overcome the limited T cell recruitment combinatorial approaches with direct or indirect microenvironment modifiers such as BRAF, MEK, or PARP inhibitors or VEGF-targeting antibody- or inhibitor-based therapies have already been evaluated (150).

Peptide-based cancer vaccines are in generally well tolerated with only a few side effects including local reaction on the vaccination site (70, 134). In a meta-analysis including 500 patients, only 1.2% of the vaccinated patients suffer from vaccine-related serious adverse events (151). Augmented immune responses seem to be involved as both cellular and humoral responses to the vaccine peptides are boosted in affected patients. Beside allergic reaction against the vaccine, a major potential risk that can be anticipated for patients treated with peptide-based vaccines is autoimmunity induced by immune reactions against normal tissues expressing the vaccinated targets at considerable levels. Therefore, a precise characterization of the target expression on normal tissues is of paramount importance. However, such autoimmunity events are very rare. In the phase I to III clinical trials of the off-the-shelf multipeptide vaccine IMA901 for the treatment of renal cell carcinoma with a total of 302 vaccinated patients (70, 134) as well as in the phase I/II trial of an antiprostate cancer vaccine treating 19 patients (152) no evidence of autoimmunity was observed.

To provide a maximum of safety for vaccinated patients and to ensure early notice of side effects, especially allergic reactions or autoimmunity, risk mitigation measures must be implemented in each clinical trial. First, patients should be monitored and kept under medical supervision for at least 2 h following each vaccination, including close monitoring of vital parameters (pulse, blood pressure, temperature, oxygen saturation) and subjective well-being, as allergic reactions were reported to occur mostly in the first hour after vaccination (71, 134). In case of severe anaphylactic reactions, standardized medical antiallergic treatment such as antihistamine should be applied. Furthermore, special emphasis should be put on any sign of the development of autoimmune disease. Any unclear inflammation should be followed up closely until it resolves, and pausing or stopping of vaccinations and immunosuppressive treatment should be considered.

## Conclusion

Antigen-specific T cell–based immunotherapy, including therapeutic peptide-based vaccination approaches, is considered one of the most promising strategies for future antitumor therapy. Although all efforts in recent decades to develop clinically effective therapeutic peptide vaccines for the treatment of patients with cancer have not yet achieved a clinical breakthrough, the constantly growing knowledge gives rise to new hope for the development of improved and optimized clinically effective future peptide vaccines. The previous lack of success is due to several so far unmet issues and prerequisites that have been identified in the last years. The perfect peptide vaccine of the future—from our point of view—should include multiple peptides of different antigens combining CD4^+^ with CD8^+^ T cell epitopes as well as mutated and unmutated tumor-associated peptides. The natural presentation of the vaccine peptides should be verified using mass spectrometry–based immunopeptidomics. Furthermore, the vaccination should be performed in an optimal effector to target cell ratio setting using a strong immune-stimulating adjuvant. The combination of peptide-based vaccines with drugs that support T cell response and the presentation of tumor-associated antigens in the immunopeptidome might further improve clinical outcome. Further progress and developments will hopefully reduce the costs and production time of partly or even fully personalized therapeutic peptide vaccination approaches enabling personalized therapy concepts for each individual cancer patient.

## Conflict of interest

A. N. and J. S. W. hold patents on peptides. H.-G. R. holds patents on peptides and adjuvants.
